# miRNA Sequence Analysis in Patients With Kaposi’s Sarcoma-Associated Herpesvirus

**DOI:** 10.3389/pore.2022.1610055

**Published:** 2022-01-24

**Authors:** Seref Bugra Tuncer, Betul Celik, Demet Akdeniz Odemis, Seda Kılıc Erciyas, Ozge Sukruoglu Erdogan, Mukaddes Avsar, Gozde Kuru Turkcan, Hulya Yazici

**Affiliations:** ^1^ Department of Cancer Genetics, Istanbul Faculty of Medicine, Oncology Institute, Istanbul University, Istanbul, Turkey; ^2^ Health Services Vocational School of Higher Education, T.C. Istanbul Aydin University, Istanbul, Turkey; ^3^ Department of Molecular Biology and Genetics, Faculty of Arts and Sciences, Halic University, Istanbul, Turkey; ^4^ Department of Medical Biology and Genetics, Arel Medical Faculty, Istanbul Arel University, Istanbul, Turkey

**Keywords:** prognosis, diagnosis, Kaposi’s sarcoma, Virus, KSHV, miRNA-seq

## Abstract

MicroRNAs (miRNAs) are the non-coding RNAs that can both attach to the untranslated and coding sections of target mRNAs, triggering destruction or post-transcriptional alteration. miRNAs regulate various cellular processes such as immune function, apoptosis, and tumorigenesis. About 35,000 miRNAs have been discovered in the human genome. The increasing evidence suggests that the dysregulation of human miRNAs may have a role in the etiology of some disorders including cancer. Only a small sub-set of human miRNAs has functionally been validated in the pathogenesis of oncogenic viruses such as Kaposi’s sarcoma-associated herpesvirus (KSHV). KSHV is the cause of various human malignancies including primary effusion lymphoma (PEL) and Kaposi’s sarcoma (KS), which are mainly seen in AIDS patients or other immunocompromised people. We aimed to identify the miRNAs in Kaposi’s sarcoma cases, with the comparison of KSHV seropositive and seronegative tumors with the controls and in each other in Turkish Kaposi’s sarcoma patients. We performed the miRNA-sequencing at genome level in the peripheral blood mononuclear cells of 16 Kaposi’s sarcoma patients, and in 8 healthy controls matched for age, gender, and ethnicity. A total of 642 miRNA molecules with different expression profiles were identified between the patients and the healthy controls. Currently, out of 642 miRNAs, 7 miRNAs (miR-92b-3p, miR-490-3p, miR-615-3p, miR-629-5p, miR-1908, miR-3180, miR-4433b-3p) which have not been described in the literature in the context of Kaposi’s sarcoma were addressed in the study for the first time and 9 novel miRNAs, not found previously in the database, have been detected in Kaposi’s sarcoma using the miRNA-sequencing technique. This study demonstrates the identification of differently expressed miRNAs which might be the new therapeutic targets for Kaposi’s sarcoma, that has limited treatment options and can be used in the etiology, diagnosis, and prognosis of this cancer.

## Introduction

MicroRNAs (miRNAs) are the single-stranded small (19–25 nucleotides long) non-coding RNA molecules, which play a role in various biological processes (1, 2). miRNAs regulate the target gene silencing by binding to complementary sequences of mRNA in the 3′-untranslated regions (3, 4) and participate in cell death, proliferation, differentiation, and signal transduction (5). Oncogenic/tumor suppressor miRNAs, as well as those involved in proliferation, angiogenesis, and other cellular processes, play an essential role in cancer (6) and consequently, miRNAs can be used as diagnostic and therapeutic response indicators.

Kaposi’s sarcoma is a type of cancer that develops from the lining cells of blood or lymph vessels. The tumors (lesions) usually occur on the skin, however, may commonly manifest particularly in the mouth, gastrointestinal system, and respiratory tract. The skin lesion manifests with red, purple, brown, and black colored spots (7). The disease which mainly affects the elderly Italian, Jewish, or Mediterranean men, was first described by Moritz Kaposi in 1872 (8, 9). Globally, Kaposi’s sarcoma is a rare type of cancer but endemic to Southern, and Eastern Africa. The disease was the most frequent cause of cancer incidence and mortality among men in Mozambique and Uganda in 2020. The highest number of Kaposi’s Sarcoma patients were detected in men in Mozambique, and women in Zambia (10).

Viruses are the cause of 12% of human cancers. Viral oncogenesis generally results from the expression of viral oncogenes (11). Many studies have shown that Human herpesvirus-8 (HHV-8), also known as Kaposi’s sarcoma-associated herpesvirus (KSHV) infection is the underlying cause of Kaposi’s sarcoma (12). Normally, the KSHV infection is controlled by the immune system and does not cause any symptoms in healthy people. However, KSHV can result with Kaposi’s sarcoma in individuals with compromised immune systems. In the Turkish population, KSHV seroprevalence is quite smaller and Kaposi’s sarcoma is commonly detected in HIV-negative individuals (13). The pathogenesis of Kaposi’s sarcoma in this population remains unclear due to the lack of genomics studies.

In particular, the geography, environmental factors, and ethnicity can result with the presence of the different features of Kaposi’s sarcoma worldwide that can be characterized from the miRNA expression profile of the peripheral blood mononuclear cells in Kaposi’s sarcoma patients. To identify the diagnostic and therapeutic targets for Kaposi’s sarcoma, we investigated the different expression profiles of miRNAs in the peripheral blood mononuclear cells of 16 Kaposi’s sarcoma patients and of 8 healthy individuals since the miRNAs produced by malignant cells appear in the circulation, and may have diagnostic or prognostic value.

We aimed to detect miRNAs in association with KSHV and correlated the data with healthy controls and KSHV seronegative state. Therefore, the KSHV seropositive patients were compared with the KSHV seronegative Kaposi’s sarcoma patients, and the healthy group. BLAST was performed over miRBase to detect existing or new miRNA sequences. The exact match of analyzed sequences with the miRNA sequences in the miRBase database in terms of nucleotide sequence and length showed that the related sequence was accepted as miRNA. This study demonstrates the identification of miRNA molecules that might be new therapeutic targets for Kaposi’s sarcoma which has limited treatment options and can be used in the etiology, diagnosis, and prognosis of this cancer.

## Materials and Methods

### Patient Collection

The study was approved by the Ethics Board of Istanbul University (Approval no. 196, dated February 19, 2017) following the Declaration of Helsinki (14). The peripheral blood mononuclear cells of 16 Kaposi’s sarcoma patients, who presented to our clinic between 2017 and 2019, and of 8 healthy individuals matched for age, sex, and ethnicity with the patients and with no history of cancer in the family for 3 generations were investigated in the study. The patients’ data for viral etiologic factors such as HIV, KSHV, HBsAg, ANTI-HBc, ANTI-CMV, EBV VCA, HHV-1, HHV-2, ANTI-VARI ZOS were identified from the patient files, and the same etiologic factors were questioned in the healthy control group.

The miRNA samples obtained from leukocytes were analyzed using the miRNA-sequencing method, and the differently-expressed miRNAs in KSHV seropositive samples compared to miRNAs in KSHV seronegative samples and healthy controls were identified. Nine Kaposi’s sarcoma patients were male, and 7 were female in the study. The mean age for men was 68 years, and 70 years for women, respectively. Five people in the control group were male and their average age was 66 years, while 3 were female and the average age was 70 years.

### Leukocytes Isolation

First, 20 ml of peripheral blood mononuclear cells of the patients were collected into a tube containing 3 ml Ficoll (Sigma-Aldrich, Darmstadt, Germany) solution. The peripheral blood mononuclear cells were diluted using phosphate-buffered saline. Phosphate-buffered saline diluted peripheral blood mononuclear cells were centrifugated at room temperature for 30 min at 1910 RPM. Cells were frozen for 24 h at −80°C, and then removed for long-term storage in the liquid nitrogen tank.

### miRNA Extraction

miRNA isolation procedure was performed using the miRNeasy Mini Kit (Qiagen, cat No./ID: 217004) following the kit protocol. The maximum sample amount used was 1 × 10^7^ cells according to the kit protocol. 700 µl QIAzol solution was added on leukocytes and vortexed. For the complete fractionation of the nucleoproteins, the leukocytes were kept at 24°C for 5 min 140 µl chloroform was added to the fractionated leukocytes, and incubated for 2–3 min at 24°C, then mixture of chloroform and fractionated leukocytes were centrifugated at +4°C at 12.000 g for 15 min. The supernatant was transferred into the collection tube and vortexed by adding 525 µl of 100% Ethanol and then 700 µl mixture was loaded on an RNeasy MiniElute spin column inserted in a 2 ml collection tube. The centrifugation process was repeated by adding 700 µl RWT and 500 µl RPE buffers to the spin columns, respectively. The columns inserted in 2 ml clean tubes were dried for 1 min. The columns inserted in 1.5 ml sterile tubes were centrifuged at 8000 g by addition of 50 µl purified water for 1 min, and the miRNAs were collected.

### miRNA Quantification and Quality Control

The quality of the miRNAs was screened by electrophoresis at 145 V in 1.5% Agarose gel. Then, the purity and concentrations of miRNAs were measured on Thermo Scientific NanoDrop 2000 device (spectrophotometer NanoDrop Technologies, Wilmington, DE, United States). The measurement rate between 260/280 nm wavelengths was the indicator of the quality of the purity of the samples, and the samples in the ideal range of 1.8 and 2.2 were included in the study. The adequacy of miRNAs for NGS analysis were evaluated for the second time on bioanalyzer device (2100 Bioanalyzer, Agilent Technologies, Santa Clara, CA, United States), using the Agilent RNA 6000 Nano Kit (Agilent Technologies, Santa Clara, CA, United States).

### miRNA Library Preparation

Twenty four miRNA samples of the healthy controls and patients were investigated using the Illumina miRNA sequencing system. The Illumina miRNA Library Preparation kit (RS-200-0012) was used in the library preparation step. The Illumina adapters used in the library preparation step directly and specifically attached to miRNAs from the 5′-phosphate and 3′-hydroxyl groups located at the endpoints of miRNAs. Single chain cDNA was obtained with the reverse transcription procedure after attachment.

#### Adapter Attachment

##### 3′ Adapter Attachment

1 µl RA3 and 5 µl RNA nuclease-free water were mixed in a 200 µl PCR tube on ice and put on a thermal cycler at 70°C for 2 min and then were placed on ice. 2 µl HML+ 1 µl RNase Inhibitor, and 1 µl T4 RNA Ligase 2 were added into 200 µl PCR tube containing 1 µl RA3 and 5 µl RNA nuclease-free water and 4 µl of this mixture was added into a RA3/total RNA containing tube and put on a thermal cycler at 28°C for 1 h. After removing from the thermal cycler, 1 µl STP was added into tube containing 1 µl RA3 + 5 µl RNA nuclease-free water + 2 µl HML + 1 µl RNase Inhibitor, and 1 µl T4 RNA Ligase 2 + RA3/total RNA and this mixture were incubated at 28°C for 15 min.

##### 5′ Adapter Attachment

In another 200 µl PCR tube, 1.1 × N μl RA5 was added and incubated at 70°C for 2 min on a thermal cycler and then placed on ice. 1.1 × N μl 10 mM ATP was added to the 200 µl PCR tube containing 1.1 × N μl RA5 and then 1.1 × N μl T4 RNA Ligase was added to the 200 µl PCR tube containing RA5/ATP mixture. 3 µl of RA5/ATP/T4 RNA Ligase solution were added into tube containing 3 µl RA3 mixture, and incubated at 28°C for 1 h.

##### Reverse Transcription

6 µl of Adapter-ligated RNA (3 µl RA5 + 3 µl RA3) library was added to 200 µl PCR tube and 1 µl RNA RT Primer was added, then mixture centrifuged and incubated on a thermal cycler at 70°C for 2 min. In another 200 µl PCR tube on ice, 2 µl of 5X First-Strand Buffer + 0.5 µl dNTP Mix + 1 µl of 100 mM DTT + 1 µl RNase Inhibitor + 1 µl SuperScript II Reverse Transcriptase were mixed well and 5.5 µl of this mixture added to the tube containing the adapter-ligated RNA/primer and then mixture centrifuged and incubated on thermal cycler at 50°C for 1 h.

##### Library Amplification

25 µl pure water + 25 µl PML + 2 µl RP1 + 2 µl RPIX were added and PCR master mix was prepeared in 200 µl PCR tube. 37.5 µl PCR master mix was added to the adapter-ligated RNA mixture tube. The following program was set at 98°C for 30 s, 11 cycles:

At 98°C, for 10 s
At 60°C, for 30 s
At 72°C, for 15 s
At 72°C, for 10 min
4°C hold

#### Purification of cDNA

##### Pellet Dilution

0.2 µl of 1X Pellet Paint NF Co-Precipitant and 1.8 µl Ultrapure water was added in a 0.1X Pellet Paint tube, then mixture centrifuged

##### Gel Electrophoresis

2 µl CRL and 2 µl DNA loading dye were mixed well in a 1.5 ml microcentrifuge tube. In another 1.5 ml microcentrifuge tube, 1 µl HRL and 1 µl DNA loading dye were mixed. 50 µl of amplified cDNA and 10 µl DNA loading dye were mixed in 1.5 ml microcentrifuge tube. Gel lanes were loaded with 2 µl CRL/loading dye mixture, 2 µl HRL/loading dye mixture, and 25 µl each of amplified cDNA/loading dye mixture. The gel was run for 60 min at 145 V.

### miRNA-Sequencing

The sequencing was performed using the Illumina NextSeq500 new generation sequencing platform. In the Solexa sequencing method used by Illumina, the DNA fragments were first inserted on chips called Flow-Cell and were regionally reproduced for providing high accuracy signal on the located region (Bridge-Amplification) and millions of fragment clusters were formed on the Flow-Cell. The fragment clusters formed on Flow-Cell were sequenced to read 1 base in each cycle. DNA fragments were marked with 2 different strains in each cycle using the “Sequencing-by-Synthesis” method developed by Illumina. The image of the cluster was taken in each cycle with the fluorescence camera following the attachment to the appropriate di-nucleotide. The images received from each cluster were combined, and finally, the nucleotide sequences of the related clusters were obtained from the radiation characteristics. The efficacy of the sequencing study was measured by investigating the FASTQ formation for detection of the location, and radiation characteristics of the DNA clusters on Flow-Cell and the quality control procedures, and raw FASTQ data. Reading filters were formed, and the low-quality base readings in FASTQ reading data were removed from these results.

### Data Filtering

Sequencing platform reading data was collected at the end of the miRNA sequencing analysis. FASTQC program, quality filters, and Trimmomatic application for cutting operations software programs were used to delete unwanted data. BLAST was performed over miRBase to detect existing or new miRNA sequences. If the analyzed sequences matched exactly with the miRNA sequences in the miRBase database in terms of nucleotide sequence and length, the related sequence was accepted as miRNA. The single sequences detected for identifying the miRNA sequences using the miRBase database were paired by performing BLAST from the miRBASE database (15). The related sequence was accepted as a miRNA in case of detection of the complete match of the miRNA sequences and nucleotide array and length in the investigated sequences miRBASE database and the miRNAs, have not been described earlier and not found in the databases were revealed.The target gene prediction was identified using the data of different databases including, miRbase, miRanda, DIANA Tools, and TargetScan.

### Statistical Analysis

**FIGURE 1:**
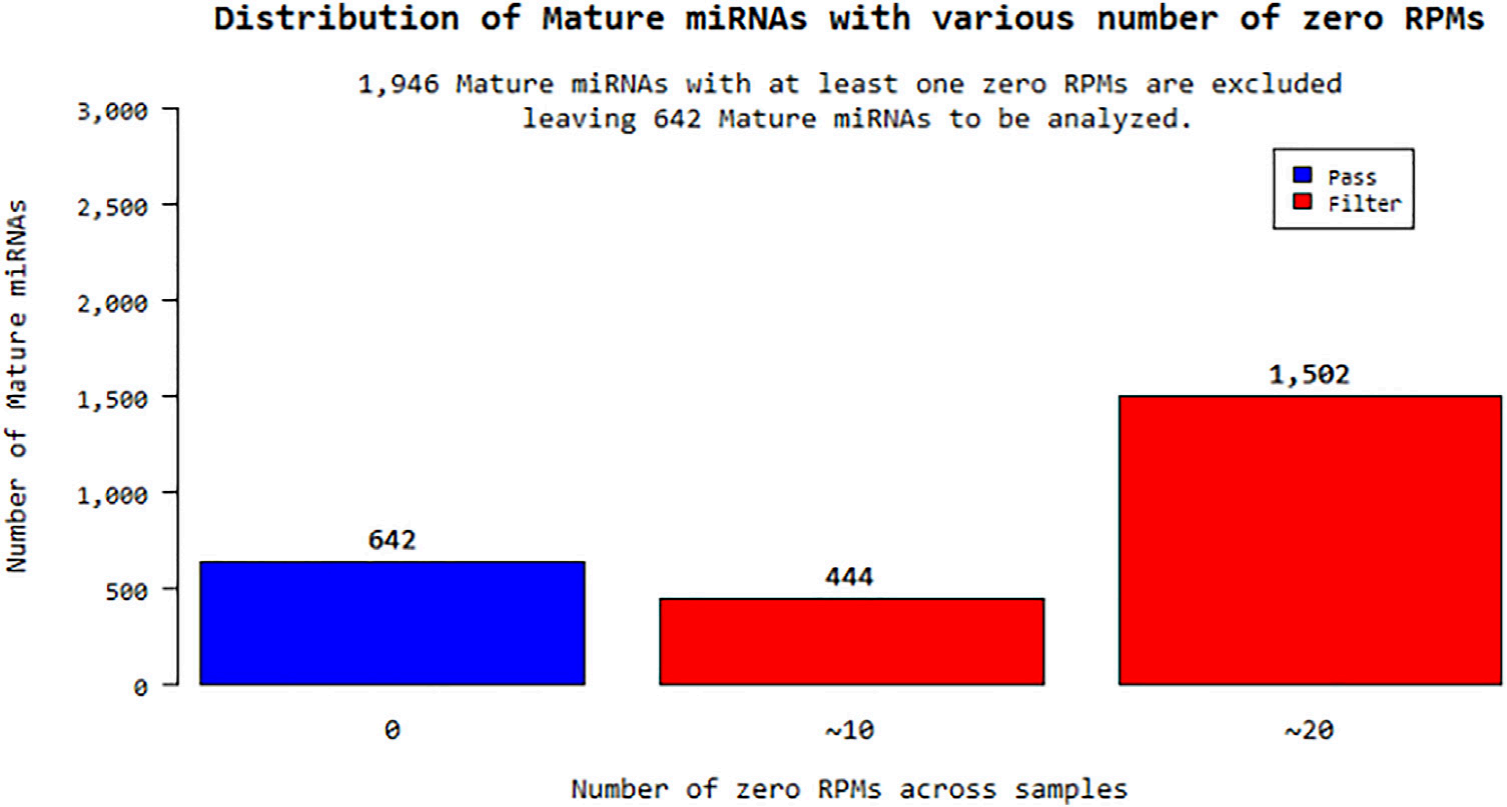
Distribution of mature miRNAs with various number of zero RPMs.

According to the fold change values of miRNAs, their significantly increased or decreased miRNAs were evaluated. miRNA sequences with an RPM (reads per million) “0” value for more than one sample for a total of 24 samples were excluded from the analysis. In this context, 1,946 out of a total of 2,588 different mature miRNAs were eliminated and statistical studies were performed on the remaining 642 miRNAs shown in Figure 1. Expression distribution of each sample was determined as a boxplot, and the percentile data (median, 50 percentile, 75 percentile, maximum and minimum) was used in the distribution. The raw distribution data log2 is converted and Quantile is normalized. Then, the intensities of miRNA expression values were evaluated as raw intensity, Log2 transformed intensity, and intensity after quantile normalization. The similarity value between the samples was calculated over the Pearson’s coefficient of log2 (RPM+1). Among the values between −1 and 1, the similarity between the samples increases as it gets closer to 1. High expression similarity was grouped according to the log2(RPM+1) value of each sample. A hierarchical clustering graph was created for the obtained groupings. The high expression similarities were grouped on the 2D graph under the log2(RPM+1) value of each sample. Samples with similar expression patterns were evaluated together. Fold Change, Independent T-test, and Hierarchical Clustering analysis methods were used. The fold change range accepted for significant results was determined as (FC ≥ 1.5), and raw *p* < 0.05.

## Results

We performed the genome level miRNA-sequencing in the peripheral blood mononuclear cells of 16 Turkish Kaposi’s sarcoma patients that are HIV negative and 8 healthy controls, who were matched in terms of age, gender, and ethnicity with the patients. In this study, the viral agent distribution of the viruses, Human Immunodeficiency Virus(HIV), Kaposi’s sarcoma-associated herpesvirus (KSHV), Hepatitis B surface antigen (HbsAg), Hepatitis B Core Antibody (ANTI-HBc), Anticytomegalovirus (ANTI-CMV), Epstein-Barr virus viral-capsid antigen (EBV VCA), Human herpesvirus 1 (HHV-1), Human herpesvirus 2(HHV-2), and Varicella-Zoster Virus (ANTI-VARI ZOS) were taken from the files of the Kaposi’s sarcoma patients. The virus infection rates of patients and healthy controls are shown in Table 1.

**TABLE 1:** Viral agent distribution in Kaposi’s Sarcoma patient group and control group.

Tests	Patients (n:16)	Positivity (%)	Healthy controls (n:8)	Positivity (%)
HIV (+)	0/16	0%	0/8	0%
KSHV (+)	8/16	50%	0/8	0%
HBsAg (+)	11/16	68%	1/8	12,5%
ANTI-HBc (+)	7/16	44%	0/8	0%
ANTI-CMV (+)	15/16	94%	4/8	50%
EBV VCA (+)	16/16	100%	4/8	50%
HHV-1 (+)	16/16	100%	4/8	50%
HHV-2 (+)	0/16	0%	0/8	0%
ANTI-VARI ZOS (+)	16/16	100%	6/8	75%

**HIV**, Human Immunodeficiency Virus; KSHV, Kaposi’s sarcoma- associated herpesvirus; HbsAg, Hepatitis B surface antigen; ANTI-HBc, Hepatitis B Core Antibody; ANTI-CMV, Anticytomegalovirus; EBV VCA, Epstein–Barr virus viral-capsid antigen; **HHV**-1, Human herpesvirus 1; HHV-2, Human herpesvirus 2; ANTI-VARI ZOS, Varicella-Zoster Virus Antibody.

The raw results of a total of 24 miRNA samples were obtained by miRNA-sequencing in the analysis. The total number of readings surpassed the Q20 and Q30 reading quality thresholds. For the raw read data, quality filtering was applied. miRNAs that are compatible with the miRBase database and their distribution have been determined after sequencing. Within the scope of the study, the sequencing results of a total of 16 patients, 8 KSHV seropositive and 8 KSHV seronegative, and the sequencing results of 8 KSHV seronegative healthy controls were investigated using biostatistical analysis after the necessary quality control, and filtering procedures were performed. Reads with an RPM (Reads Per Million) value of more than one were excluded from miRNA analysis. Of the 2,588 different mature-miRNAs found in the total, 1,946 were excluded and the remaining 642 miRNAs were statistically analyzed.

In this study, we aimed to detect differently expressed miRNAs in association with KSHV seropositive patients and KSHV seronegative patients and correlated the data with healthy controls. Therefore, KSHV seropositive patients were compared with the healthy controls (shown in Supplementary Table S1), KSHV seronegative patients and healthy control were compared (shown in Supplementary Table S2), all KSHV patients were compared with healthy controls (shown in Supplementary Table S3), KSHV seropositive patients were compared with KSHV seronegative patients (shown in Supplementary Table S4).

**FIGURE 2:**
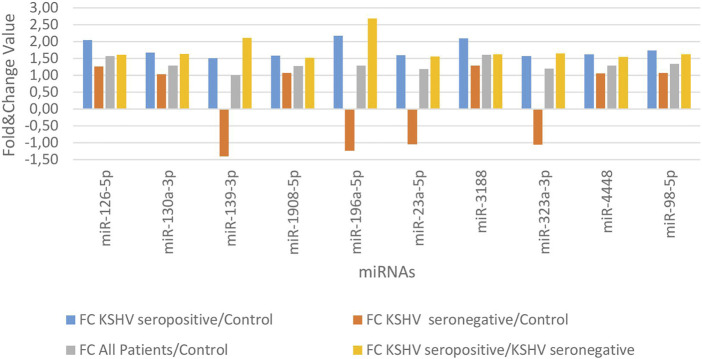
The high-expression profile of miRNAs associated with the KSHV seropositive Kaposi Sarcoma.

**FIGURE 3:**
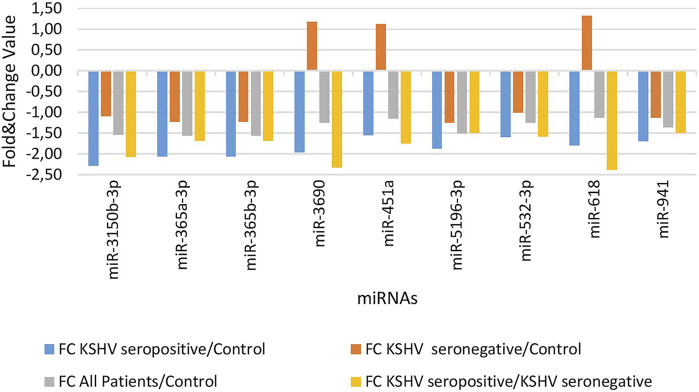
The low-expression profile of miRNAs associated with the KSHV seropositive Kaposi Sarcoma.

**FIGURE 4:**
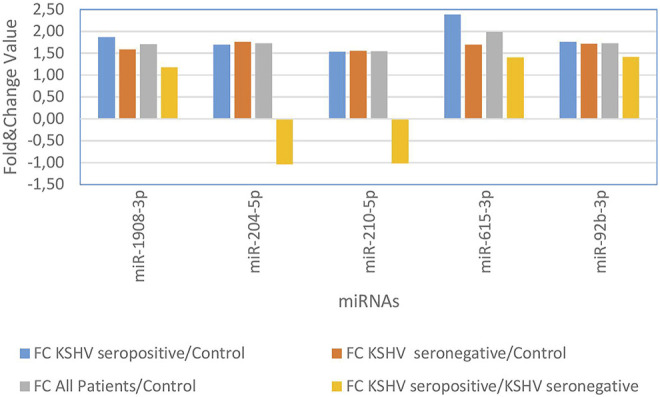
The high-expression profile of miRNAs associated with Kaposi Sarcoma.

**FIGURE 5:**
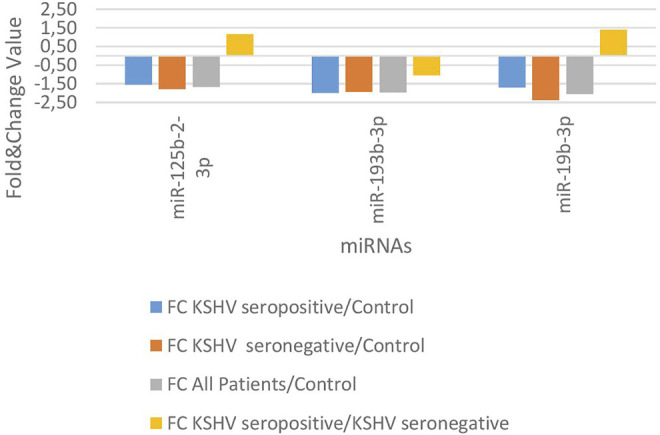
The low-expression profile of miRNAs associated with Kaposi Sarcoma.

**FIGURE 6:**
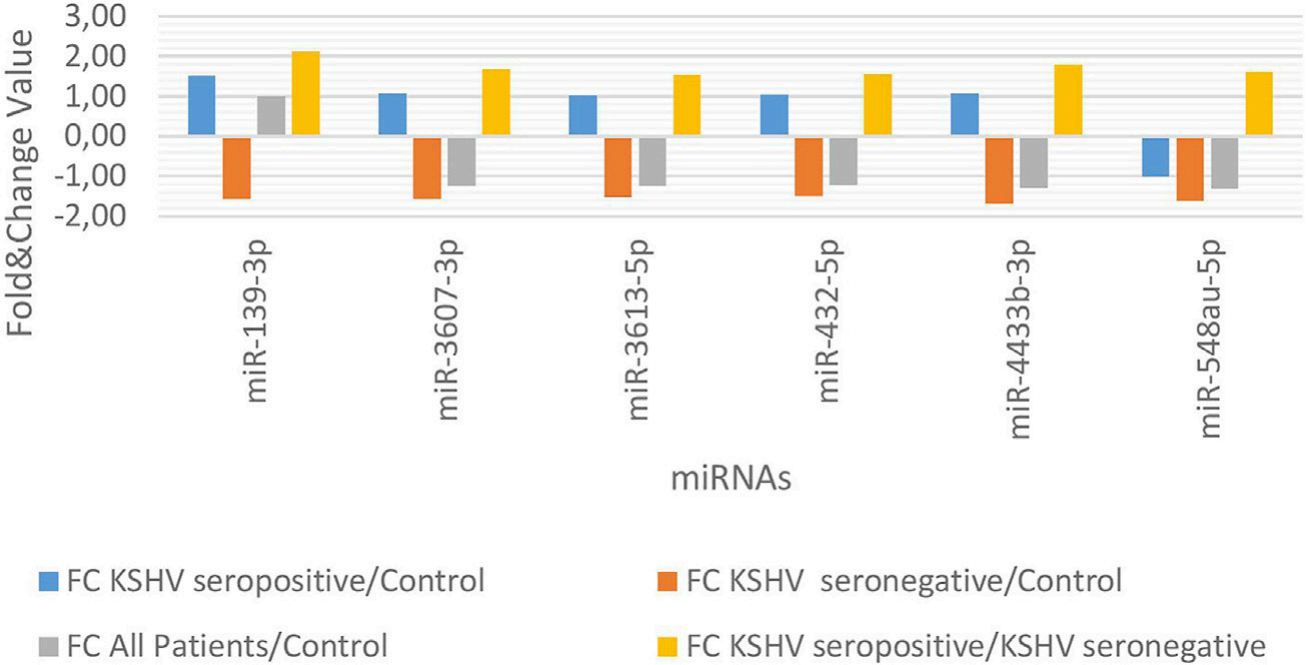
The high and low expression profile of miRNA associated with the KSHV seronegative Kaposi Sarcoma.

KSHV seropositive patients were compared to KSHV seronegative patients and healthy controls separately. The miRNAs shown an increase (Figure 2) and a decrease (Figure 3) in both comparisons were selected and shown. KSHV seropositive patients, KSHV seronegative patients, and all Kaposi’s sarcoma patients were compared to healthy controls separately. After these comparisons, the miRNAs that showed an increase (Figure 4) and a decrease (Figure 5) in all groups were selected and shown. Figure 6 represents the decreased expression profile of miRNAs of KSHV seronegative patients compared to the expression profile in both KSHV seropositive patients and healthy control separately. The fold change range accepted for significant results was determined as (FC ≥ 1.5), and raw *p* < 0.05 for Figures 2–6.

### 9 miRNAs (Not Existing in Any Database)

Furthermore, a total of 967 miRNAs were discovered after the miRNA-sequencing technique. 840 out of 967 miRNAs were removed after filtering, and quality control measures. The statistical analysis of 127 miRNAs was carried out with separate t-tests. The presence of 9 novel miRNAs, which were not previously recorded in the miRBase and RFAM database but whose expression was substantially increased by *p*-value (*p* < 0.05) and Fold-Change (FC ≥ 1.5) were calculated. Four miRNAs (miRNAs 1,2,3,4) that were found to have shown an increase in expression were discovered after the comparison of KSHV seropositive patients, and the healthy controls. The 3 miRNAs (miRNAs 5,6,7) showed an increased expression after the comparison between KSHV seronegative patients, and the healthy controls. Furthermore, 2 miRNAs (miRNAs 8,9) showed an increased expression both in KSHV seropositive and KSHV seronegative patients. 9 novel miRNA sequences, not found previously in the database, are shown in Table 2.

**TABLE 2:** Nine Novel miRNAs (not found previously in the database) in KSHV patients.

Candidate miRNA	Chr	miRNA sequences	(FC) Value	*p*-Values
1. Novel miRNA	1	AGU​UGA​AUC​UCU​GAA​UAG​ACC​AAU​AAC​AGG​CUC​UGA​AA	2,04	0,0116 (KSHV+/HC)
		UUG​UGG​CAA​UAA​UCA​AUA​G		
2. Novel miRNA	8	GAG​GCA​UAA​AUG​CAG​AUU​UUU​UUU​UUC​CUC​CAG​UGA​AU	1,73	0,0354 (KSHV+/HC)
		UUU​CUG​UAA​CCA​UGG​GCC​UCG​CUU​UAA		
3. Novel miRNA	12	AGC​CAU​AAA​UCG​GCA​CAG​AAA​GCA​AUG​CAU​AUU​AAG​GG	1,64	0,0488 (KSHV+/HC)
		GGC​GGA​UGC​UGG​ACA​UGG​CUC​GGG​GGC​U		
4. Novel miRNA	12	GAG​GUU​UUA​CCU​CUU​UAU​CUU​GUG​AGU​AAU​GUU​CAC​AA	2,37	0,0022 (KSHV+/HC)
5. Novel miRNA	19	CUG​CCA​UCU​GCU​GGG​AAG​UUG​UAA​UAA​UAC​AAA​UAU​CC	1,50	0,0198 (KSHV-/HC)
		AUA​CAC​GAU​GGC​UAG​GAU​GU		
6. Novel miRNA	18	GCU​CGA​GGU​GGC​GGA​GGG​CGG​AGG​CGA​GGC​CCG​CGG​GC	1,59	0,019 (KSHV-/HC)
		CCU​CUC​CCU​CCU​CCA​CCU​CCU​CGU​CC		
7. Novel miRNA	22	AUC​UAC​AGG​GCG​GCC​UCC​ACA​AAG​CUG​CCA​UUA​CAA​UC	1,96	0,006 (KSHV-/HC)
		UGA​UGG​CAG​AUA​AAG​CAG​GCC​C		
8. Novel miRNA	1	CUC​GUU​UCG​AGG​GAC​UGA​GCC​CCC​UAC​AAC​GAC​ACC​GG	1,66	0,044 (KSHV(+,-)/HC)
		CUG​GUC​CAC​GCA​GCG​CUG​GCC​GA		
9. Novel miRNA	10	UAU​GGU​CUA​UAA​AGU​CAC​UGU​GAA​CAG​UGA​AUU​AGC​AA	2,17	0,023 (KSHV(+,-)/HC)
		AGA​CUG​CAC​CAU​UUC​UCC​GAG​GGA​UCA​AGU		

Chr, Chromosome; FC, Fold & Change; KSHV+, Kaposis’sarcoma seropositive; KSHV -, Kaposi’s sarcoma seronagtive; HC, healthy control.

## Discussion

Currently, Herpes viruses that infect humans are known to encode their own miRNAs (15). KSHV is one of the best examples of viruses encoding their miRNAs which was identified in several studies (16, 17). Research has focused on the role of these miRNAs in KSHV infection and pathogenesis after it was found that KSHV encodes specific miRNAs (18). The expression of viral, and cellular genes required for disease-associated virus infection was shown to be regulated by KSHV-specific miRNAs (19, 20). These miRNAs were also found to have been highly expressed in the latent phase of virus infection (21). The KSHV virus has been reported to significantly change the expression of several cellular miRNAs, in addition to encoding the specific miRNAs (22, 23). miRNAs have been shown to control the entry of KSHV into the cell, its replication, pathogenesis, and the escape of the immune system from the virus (22, 23). Recent studies have indicated that the expression differences of miRNAs have many roles in the initiation, and progression of various cancers. As a consequence, the detection of molecules that may be the new biological markers is very significant in terms of diagnosis, and treatment.

According to literature, miRNAs with various expression profiles have been identified in the obtained tissue and peripheral blood mononuclear cells of Kaposi’s sarcoma patients (5). The miRNAs miR-126-3p, miR-199a-3p, and miR-16-5p were found to have a high level of expression (5). Our research showed that miR-126-3p and miR-126-5p displayed an increased expression profile in KSHV seropositive patients.

Wang et al. noted that the increased expression of other miRNAs mediating from epithelium to the mesenchymal transition, the increased expression of miR-92b-3p in the gastric cell line accelerated cell adhesion and invasion of tumor metastasis through the PI3K/Akt signaling pathway (24). In compliance with other studies, we found that the level of miR-92b-3p expression increased in Kaposi’s sarcoma.

Miyamoto et al. used mouse hepatocyte cell lines, primary mouse hepatocytes, and human hepatocellular carcinoma lines, and found miR-615 differences in five different cell lines, and, emphasized that miR-615 regulates lipoapoptosis by inhibiting the C/EBP homologous protein (25). Zhang et al. in their study indicated that miR-490 was upregulated in hepatocellular carcinoma tissues compared with the miR-490 in normal tissue. Researchers determined that the miR-490 overexpression leads to an increase in cell proliferation, cell migration, and invasion and indicated that this accelerated the epithelial-mesenchymal transformation. Experimentally, miR-490-3p inhibition was shown to have the opposite effect on cell lines (26). In our study, the expression levels of miR-615-3p and miR-490-3p were found to have increased in Kaposi’s sarcoma patients.

The upregulation of miR-1908 in osteosarcoma and glioblastoma tissues were closely linked to cell proliferation, invasion, tumor growth, and advanced tumor staging (27, 28). The miR-1908 was shown to provide this effect by suppressing the PTEN expression (27, 28). We also found the higher expression profile of miR-1908 in Kaposi’s sarcoma patients in the present study.

In addition, miR-210 is associated with tumor hypoxia, and accepted as a biomarker has been reported to be mostly regulated by miRNA in lung cancer (29). Xie et al. in particular suggested that miR-210 has been shown to have prognostic value in breast cancer patients (30). Tang et al. emphasized in another study that miR-210 up-regulation was associated with poor prognosis in acute myeloid leukemia and may be useful as a prognostic marker to predict the clinical outcome of patients with AML (31). We found an increased miR-210 expression level in Kaposi’s sarcoma patients in our study.

The higher miR-191 expression level was found in cancer tissues of patients in the literature, and that its high pattern of expression played a role in aggressive tumor proliferation, and progression (32–34). Here in this study, miR-191 was found to have increased in KSHV seronegative Kaposi’s sarcoma patients.

In our research, the miR-4433b-3p showed increased expression in KSHV seropositive patients compared to seronegative patients. The increased expression of miR-4433 in nasal tissue epithelial cells could not fulfill the defensive and pathogen barrier in epithelial cells in the study of Miyata et al. (35). The increased miR-4433 expression has been reported to also inhibit the activation of cytokines that play a role in the inflammatory response (35).

We found that miR-629-5p had an increased expression in KSHV seropositive Kaposi’s sarcoma patients in our study. In another study, miR-629 showed high expression in systemic lupus erythematosus (SLE) patients.

In our study, the expression of miR-365b was found to have decreased in accordance with the expression in retinoblastoma tissues, and colon cancer in the literature (36, 37). It has been shown that miR-365b usually plays an active role in eye development and inhibits the cell cycle in the G1 stage with its tumor suppressor properties. In colon cancer, the loss of expression, and regulation of miR-365 has been shown to eliminate the tumor-suppressing effect (36, 37). In our research, it was determined that the expression levels of miR-365a and miR-365b were significantly lower in Kaposi’s sarcoma patients.

In the literature, the miR-3180 expression decreased in Kaposi’s sarcoma patients and buccal mucosa of patients with oral submucous fibrosis. The miR-3180 expression has been reported to be extremely reduced, particularly in patients with active mucosal wounds, and it was emphasized that miR-3180 functions as an immune system member (38). In this study, the miR-3180 expression level was significantly decreased in patients with Kaposi’s sarcoma.

Since the miRNAs produced by malignant cells appear in the circulation and may have diagnostic or prognostic value, we investigated the different expression profiles of miRNAs in the peripheral blood mononuclear cells of 16 Kaposi’s sarcoma patients and in 8 healthy individuals to find diagnostic and therapeutic targets for Kaposi’s sarcoma. However, the ability of miRNA molecules to be used in disease pathogenesis, prognosis, and treatment selection will be understood in studies carried out in groups with a high number of patients. Currently, some miRNA-based treatment methods are being tested in clinical trials (39). The use of miR-122 antisense oligonucleotide inhibitors is one of the most common examples of miRNA-based antiviral therapy. Thanks to this treatment process, advances have been made in the treatment of HCV infection (39). Furthermore, miRNA vaccines directly targeting the viral genome are the other treatment options. Vaccine-like transfer of tissue-specific miRNA target sequences into tissue prevents the virus from proliferating in specific tissues (40). The replication of the virus in the central nervous system was prevented by adding the targets of neuron-specific miR-124 to the poliovirus genome (41). In poliovirus-infected animals, the engineered virus strain has been shown to develop protective immunity (41). Safe and effective vaccine strains have been applied to the influenza virus using a similar methodology (42).

miRNAs activity regulates cell proliferation, cell death, and tumorigenesis, so the miRNAs may be essential in the treatment of the disease. Therefore, it has recently been proposed that miRNAs may be antiviral or therapeutic targets. In this study, we analyzed 642 miRNAs and found seven miRNAs (miR-92b-3p, miR-490-3p, miR-615-3p, miR-629-5p, miR-4433-3p, miR-3180, miR-4433b-3p) which were not previously recorded in the public databases associated with Kaposi’s sarcoma. In addition, we used the miRNA-sequencing technique and further described 9 novel miRNAs which were not previously found in the database. Future research will assess the potential of these miRNAs in the diagnosis, prognosis, and treatment of Kaposi’s sarcoma, and we suggest that they will contribute to the development of alternative treatment protocols for Kaposi’s sarcoma patients.

## Data Availability

The datasets presented in this study can be found in online repositories. The names of the repository/repositories and accession number(s) can be found in the article/Supplementary Material.
