# Supplementary material for: miRNA Sequence Analysis in Patients With Kaposi’s Sarcoma-Associated Herpesvirus
Source: Pathol Oncol Res. 2022 Jan 24;28:1610055. doi: 10.3389/pore.2022.1610055 (PMC8820206; doi:10.3389/pore.2022.1610055)
Supplement: Supplementary file 1 [file Table1.DOCX]

**Supplementary Table** **1**. Differently expressed miRNAs in KSHV seropositive Kaposi’s sarcoma patients v.s healthy controls

**miRNAs Fold Change miRNAStatus miRNAsequence Target Gene**

**(FC) Value**

| \| let-7f-5p \| 1.59 \| Upregulated \| UGAGGUAGUAGAUUGUAUAGUU \| PRDM1,KLK10,KLK6,CCND1,MYH9 \| \| --- \| --- \| --- \| --- \| --- \| \| miR-126-3p \| 1.58 \| Upregulated \| UCGUACCGUGAGUAAUAAUGCG \| TOM1,CRK,VEGFA,SOX2, TWF1 \| \| miR-126-5p \| 2.04 \| Upregulated \| CAUUAUUACUUUUGGUACGCG \| SPRED1,SLC45A3,PTPN7, MYC,ADAM9 \| \| miR-1260a \| 1.50 \| Upregulated \| AUCCCACCUCUGCCACCA \| DX1,ZNF432,PSAT1,SCD, RPS27 \| \| miR-1306-3p \| 1.59 \| Upregulated \| CCACCUCCCCUGCAAACGUCCA \| HSPA6,PCBD2,ZNF425,TRIB1,CDK4 \| \| miR-130a-3p \| 1.67 \| Upregulated \| CAGUGCAAUGUUAAAAGGGCAU \| HOXA5,PPARG,ATXN1, MEOX2,KLF4 \| \| miR-133a-3p \| 1.51 \| Upregulated \| UUUGGUCCCCUUCAACCAGCUG \| FSCN1,TAGLN2,CDC42,PNP,HCN2 \| \| miR-139-3p \| 1.50 \| Upregulated \| UGGAGACGCGGCCCUGUUGGAGU \| LRPPRC,THY1,BARHL1, MTDH,FOXC1 \| \| miR-15b-3p \| 1.56 \| Upregulated \| CGAAUCAUUAUUUGCUGCUCUA \| IGF1R,RND2,SSFA2,MUC15, STX11 \| \| miR-1908-3p \| 1.86 \| Upregulated \| CGGCGGGGACGGCGAUUGGUC \| PLD5,CASZ1,PITX1,EPB41, RPL37 \| \| miR-1908-5p \| 1.57 \| Upregulated \| CGGCGGGGACGGCGAUUGGUC \| NKIRAS2,APOE,DNAJA4, ATP6,VGF \| \| miR-196a-5p \| 2.17 \| Upregulated \| UAGGUAGUUUCAUGUUGUUGGG \| HOXC8,BACH1,CDKN1B, ANXA1,HOXA7 \| \| miR-204-5p \| 1.70 \| Upregulated \| UUCCCUUUGUCAUCCUAUGCCU \| BCL2,MEIS1,THRB,AP1S2, EZR \| \| miR-210-5p \| 1.53 \| Upregulated \| AGCCCCUGCCCACCGCACACUG \| BTG2,FBXO28,PDE7A,STX17,HIP1 \| \| miR-23a-5p \| 1.59 \| Upregulated \| GGGGUUCCUGGGGAUGGGAUUU \| CHD4,WNT7B,GSR,ATF1, HDGF \| \| miR-30d-3p \| 1.60 \| Upregulated \| UGUAAACAUCCCCGACUGGAAG \| KPNA2,EIF1,MYC,GNAL, VAV3 \| \| miR-3188 \| 2.09 \| Upregulated \| AGAGGCUUUGUGCGGAUACGGGG \| ADM,ESPL1,EGF,CTC1, ACTG1 \| \| miR-323a-3p \| 1.56 \| Upregulated \| CACAUUACACGGUCGACCUCU \| SMAD2,SMAD3,WDR45B, MYLIP,ZXDA \| \| miR-34a-5p \| 1.55 \| Upregulated \| UGGCAGUGUCUUAGCUGGUUGU \| MYC,CDK6,E2F3,MDM4, BCL2 \| \| miR-421 \| 1.51 \| Upregulated \| AUCAACAGACAUUAAUUGGGCGC \| ATM,CBX7,SMAD4,CASP3, FOXO4 \| \| miR-4448 \| 1.62 \| Upregulated \| GGCUCCUUGGUCUAGGGGUA \| CCDC88A,MRC1,CSNK1E, SEPT8,MSN \| \| miR-490-3p \| 1.96 \| Upregulated \| CAACCUGGAGGACUCCAUGCUG \| SMARCD1,PAPPA,CCND1, ABCC2,TGFBR1 \| \| miR-5189-5p \| 1.55 \| Upregulated \| UCUGGGCACAGGCGGAUGGACAG \| DCTN5,SRSF3,MRI1,ZBTB34,PSMD3 \| \| miR-615-3p \| 2.38 \| Upregulated \| GGGGGUCCCCGGUGCUCGGAUC \| LCOR,KIF14,CDC37,KLHL42, PBX3 \| \| miR-92b-3p \| 1.76 \| Upregulated \| AGGGACGGGACGCGGUGCAGUG \| SLC15A1,DAB2IP,CDKN1C, SMAD3,NLK \| \| miR-98-5p \| 1.73 \| Upregulated \| UGAGGUAGUAAGUUGUAUUGUU \| HMGA2,PGRMC1,E2F2,MYC,TUSC2 \| \| miR-1249-3p \| -1.59 \| Downregulated \| ACGCCCUUCCCCCCCUUCUUCA \| PLCG2,RBMS1,LTBP3,PAN2,KLF13 \| \| miR-125b-2-3p \| -1.54 \| Downregulated \| UCACAAGUCAGGCUCUUGGGAC \| IGF1R,HN1L,KDELC2, BCL2L11,BTG2 \| \| miR-138-5p \| -1.59 \| Downregulated \| AGCUGGUGUUGUGAAUCAGGCCG \| RHOC,ARHGEF3,ROCK2, TERT,EZH2 \| \| miR-145-5p \| -1.87 \| Downregulated \| GUCCAGUUUUCCCAGGAAUCCCU \| BNIP3,CDKN1A,STAT1, VEGFA,MYC \| \| miR-18a-5p \| -1.59 \| Downregulated \| UAAGGUGCAUCUAGUGCAGAUAG \| ESR1,SMAD4,DICER1,PTEN, ATM \| \| miR-193b-3p \| -1.99 \| Downregulated \| AACUGGCCCUCAAAGUCCCGCU \| CCND1,PLAU,MCL1,KRAS, NF1 \| \| miR-19b-3p \| -1.71 \| Downregulated \| UGUGCAAAUCCAUGCAAAACUGA \| PTEN,ATXN1,TGFBR2, BMPR2,HIPK1 \| \| miR-2467-5p \| -1.57 \| Downregulated \| UGAGGCUCUGUUAGCCUUGGCUC \| ADM,ESPL1,LAPTM4B,EGF, ACTG1 \| \| miR-296-3p \| -1.58 \| Downregulated \| GAGGGUUGGGUGGAGGCUCUCC \| KCNH1,ICAM1,TFAP2A, ZNF669,SLC7A5 \| \| miR-296-5p \| -1.54 \| Downregulated \| AGGGCCCCCCCUCAAUCCUGU \| WNK4,CDX1,HGS,BBC3, S100B \| \| miR-3150b-3p \| -2.29 \| Downregulated \| CAACCUCGAGGAUCUCCCCAGC \| ARL6IP1,MRVI1,RBM8A, STX6,HMGB3 \| \| miR-331-3p \| -1.79 \| Downregulated \| GCCCCUGGGCCUAUCCUAGAA \| ERBB2,NRP2,DOHH,HOTAIR,E2F1 \| \| miR-365a-3p \| -2.07 \| Downregulated \| UAAUGCCCCUAAAAAUCCUUAU \| CCND1,CDC25A,BCL2,KRAS,BAX \| \| miR-365b-3p \| -2.07 \| Downregulated \| UAAUGCCCCUAAAAAUCCUUAU \| SNRK,YRDC,MYLIP,UBB, MCOLN2 \| \| miR-3690 \| -1.97 \| Downregulated \| ACCUGGACCCAGCGUAGACAAAG \| CCDC71L,STX1,BRAF1,RIMS4,ZNF486 \| \| miR-378a-5p \| -1.67 \| Downregulated \| CUCCUGACUCCAGGUCCUGUGU \| SUFU,TUSC2,NODAL,SART3 \| \| miR-451a \| -1.56 \| Downregulated \| AAACCGUUACCAUUACUGAGUU \| MIF,BCL2,RAB14,AKT1, MMP2 \| \| miR-500a-3p \| -1.55 \| Downregulated \| AUGCACCUGGGCAAGGAUUCUG \| ANKEF1,KLHL15,LRRC55, VAV3,MYLIP \| \| miR-500a-5p \| -1.63 \| Downregulated \| UAAUCCUUGCUACCUGGGUGAGA \| CYLD,TAX1BP1,OTUD7B, ZNF460,GALK2 \| \| miR-5196-3p \| -1.88 \| Downregulated \| UCAUCCUCGUCUCCCUCCCAG \| H3F3C,TXNIP,GNAS,TECPR2,H3F3B \| \| miR-532-3p \| -1.60 \| Downregulated \| CCUCCCACACCCAAGGCUUGCA \| ERCC1,GAN,MFN2,F2-TMEM119 \| \| miR-618 \| -1.80 \| Downregulated \| AAACUCUACUUGUCCUUCUGAGU \| STRN,MBD2,DVL3,ZNF529, IFNAR1 \| \| miR-6803-3p \| -1.51 \| Downregulated \| UCCCUCGCCUUCUCACCCUCAG \| ZNF483,PTCD1,PTPRF,HCN2,HOXB6 \| \| miR-873-3p \| -1.55 \| Downregulated \| AGACUGGUGAGUUCCCGGGAA \| HES7,DCAF7,ABCC5,TMED7,ZNF611 \| \| miR-941 \| -1.70 \| Downregulated \| CACCCGGCUGUGUGCACAUGUGC \| KDM6B,ZNF431,HSPA6, TRPS1,CEP19 \| |  |  |  |  |  |  |
| --- | --- | --- | --- | --- | --- | --- | --- | --- | --- | --- | --- | --- | --- | --- | --- | --- | --- | --- | --- | --- | --- | --- | --- | --- | --- | --- | --- | --- | --- | --- | --- | --- | --- | --- | --- | --- | --- | --- | --- | --- | --- | --- | --- | --- | --- | --- | --- | --- | --- | --- | --- | --- | --- | --- | --- | --- | --- | --- | --- | --- | --- | --- | --- | --- | --- | --- | --- | --- | --- | --- | --- | --- | --- | --- | --- | --- | --- | --- | --- | --- | --- | --- | --- | --- | --- | --- | --- | --- | --- | --- | --- | --- | --- | --- | --- | --- | --- | --- | --- | --- | --- | --- | --- | --- | --- | --- | --- | --- | --- | --- | --- | --- | --- | --- | --- | --- | --- | --- | --- | --- | --- | --- | --- | --- | --- | --- | --- | --- | --- | --- | --- | --- | --- | --- | --- | --- | --- | --- | --- | --- | --- | --- | --- | --- | --- | --- | --- | --- | --- | --- | --- | --- | --- | --- | --- | --- | --- | --- | --- | --- | --- | --- | --- | --- | --- | --- | --- | --- | --- | --- | --- | --- | --- | --- | --- | --- | --- | --- | --- | --- | --- | --- | --- | --- | --- | --- | --- | --- | --- | --- | --- | --- | --- | --- | --- | --- | --- | --- | --- | --- | --- | --- | --- | --- | --- | --- | --- | --- | --- | --- | --- | --- | --- | --- | --- | --- | --- | --- | --- | --- | --- | --- | --- | --- | --- | --- | --- | --- | --- | --- | --- | --- | --- | --- | --- | --- | --- | --- | --- | --- | --- | --- | --- | --- | --- | --- | --- | --- | --- | --- | --- | --- | --- | --- | --- | --- | --- | --- | --- | --- | --- |

**Supplementary Table** **2.** Differently expressed miRNAs in KSHV seronegative Kaposi’s sarcoma patients v.s healthy controls

**miRNAs Fold Change miRNAStatus miRNAsequence Target Gene**

**(FC) Value**

| miR-1908-3p | 1.59 | Upregulated | CGGCGGGGACGGCGAUUGGUC | PLD5,CASZ1,PITX1,EPB41, RPL37 |
| --- | --- | --- | --- | --- |
| miR-191-3p | 1.63 | Upregulated | GCUGCGCUUGGAUUUCGUCCCC | ACTB,DMKN,MAP7D1, ZNF557,PTK6 |
| miR-204-5p | 1.76 | Upregulated | UUCCCUUUGUCAUCCUAUGCCU | BCL2,MEIS1,THRB,AP1S2, EZR |
| miR-210-5p | 1.55 | Upregulated | AGCCCCUGCCCACCGCACACUG | BTG2,FBXO28,PDE7A,STX17,HIP1 |
| miR-224-5p | 1.68 | Upregulated | UCAAGUCACUAGUGGUUCCGUUUA | CDC42,MBD2,CXCR4,API5, CDH1 |
| miR-509-3p | 1.67 | Upregulated | UGAUUGGUACGUCUGUGGGUAG | NTRK3,ZNF460,COX10, INSIG1,ZFX |
| miR-615-3p | 1.70 | Upregulated | GGGGGUCCCCGGUGCUCGGAUC | LCOR,KIF14,CDC37,KLHL42, PBX3 |
| miR-92b-3p | 1.71 | Upregulated | AGGGACGGGACGCGGUGCAGUG | SLC15A1,DAB2IP,CDKN1C, SMAD3,NLK |
| miR-125b-2-3p | -1.78 | Downregulated | UCACAAGUCAGGCUCUUGGGAC | IGF1R,HN1L,KDELC2, BCL2L11,BTG2 |
| miR-139-3p | -1.55 | Downregulated | UCUACAGUGCACGUGUCUCCAGU | CXCR4,MCL1,JUN,IGF1R, NOTCH1 |
| miR-193b-3p | -1.93 | Downregulated | AACUGGCCCUCAAAGUCCCGCU | CCND1,PLAU,MCL1,KRAS, NF1 |
| miR-19b-3p | -2.38 | Downregulated | UGUGCAAAUCCAUGCAAAACUGA | PTEN,ATXN1,TGFBR2, BMPR2,HIPK1 |
| miR-31-5p | -1.63 | Downregulated | AGGCAAGAUGCUGGCAUAGCU | RHOA,SATB2,FOXP3,MMP16,RDX |
| miR-3607-3p | -1.56 | Downregulated | ACUGUAAACGCUUUCUGAUG | MIDN,GIGYF1,SMIM15, USP42,FAM3C |
| miR-3613-5p | -1.51 | Downregulated | UGUUGUACUUUUUUUUUUGUUC | ANP32B,H3F3C,VPS13B, MYO10,MTF2 |
| miR-432-5p | -1.50 | Downregulated | UCUUGGAGUAGGUCAUUGGGUGG | MECP2,RCOR1,NES,BLCAP, LRPPRC |
| miR-4433b-3p | -1.67 | Downregulated | CAGGAGUGGGGGGUGGGACGU | DDX39B,ABCF2,UNK,RAD21,ARF1 |
| miR-485-3p | -1.61 | Downregulated | GUCAUACACGGCUCUCCUCUCU | NFYB,SLC40A1,NTRK3, KPNA2,PCGF3 |
| miR-548am-5p | -1.59 | Downregulated | AAAAGUAAUUGCGGUUUUUGCC | ANKEF1,PTBP2,SVOP,INHBA,ZNRF2 |
| miR-548au-5p | -1.62 | Downregulated | AAAAGUAAUUGCGGUUUUUGC | ANKEF1,PTBP2,SVOP,INHBA,CDK4 |
| miR-548c-5p | -1.59 | Downregulated | AAAAGUAAUUGCGGUUUUUGCC | ANKEF1,PTBP2,SVOP,INHBA,MTMR6 |
| miR-548o-5p | -1.59 | Downregulated | AAAAGUAAUUGCGGUUUUUGCC | ANKEF1,PTBP2,SVOP,INHBA,CLIP1 |
| miR-664a-3p | -1.62 | Downregulated | UAUUCAUUUAUCCCCAGCCUACA | SNRPB2,OLIG3,ZNF667, ZBTB34,RAN |

**Supplementary Table** **3.** Differently expressed miRNAs in Kaposi’s sarcoma patients v.s healthy controls

**miRNAs Fold Change miRNAStatus miRNAsequence Target Gene**

**(FC) Value**

| miR-126-5p | 1.57 | Upregulated | CAUUAUUACUUUUGGUACGCG | SPRED1,SLC45A3,PTPN7, MYC,ADAM9 |
| --- | --- | --- | --- | --- |
| miR-15b-3p | 1.58 | Upregulated | CGAAUCAUUAUUUGCUGCUCUA | IGF1R,RND2,SSFA2,MUC15, STX11 |
| miR-1908-3p | 1.71 | Upregulated | CGGCGGGGACGGCGAUUGGUC | PLD5,CASZ1,PITX1,EPB41, RPL37 |
| miR-191-3p | 1.52 | Upregulated | GCUGCGCUUGGAUUUCGUCCCC | ACTB,DMKN,MAP7D1, ZNF557,PTK6 |
| miR-204-5p | 1.73 | Upregulated | UUCCCUUUGUCAUCCUAUGCCU | BCL2,MEIS1,THRB,AP1S2, EZR |
| miR-210-5p | 1.54 | Upregulated | AGCCCCUGCCCACCGCACACUG | BTG2,FBXO28,PDE7A,STX17,HIP1 |
| miR-30d-3p | 1.58 | Upregulated | UGUAAACAUCCCCGACUGGAAG | KPNA2,EIF1,MYC,GNAL, VAV3 |
| miR-3188 | 1.61 | Upregulated | AGAGGCUUUGUGCGGAUACGGGG | ADM,ESPL1,EGF,CTC1, ACTG1 |
| miR-490-3p | 1.67 | Upregulated | CAACCUGGAGGACUCCAUGCUG | SMARCD1,PAPPA,CCND1, ABCC2,TGFBR1 |
| miR-5189-5p | 1.58 | Upregulated | UCUGGGCACAGGCGGAUGGACAG | DCTN5,SRSF3,MRI1,ZBTB34,PSMD3 |
| miR-615-3p | 1.98 | Upregulated | GGGGGUCCCCGGUGCUCGGAUC | LCOR,KIF14,CDC37,KLHL42, PBX3 |
| miR-92b-3p | 1.73 | Upregulated | AGGGACGGGACGCGGUGCAGUG | SLC15A1,DAB2IP,CDKN1C, SMAD3,NLK |
| miR-125b-2-3p | -1.66 | Downregulated | UCACAAGUCAGGCUCUUGGGAC | IGF1R,HN1L,KDELC2, BCL2L11,BTG2 |
| miR-145-5p | -1.64 | Downregulated | GUCCAGUUUUCCCAGGAAUCCCU | BNIP3,CDKN1A,STAT1, VEGFA,MYC |
| miR-193b-3p | -1.95 | Downregulated | AACUGGCCCUCAAAGUCCCGCU | CCND1,PLAU,MCL1,KRAS, NF1 |
| miR-19b-3p | -2.04 | Downregulated | UGUGCAAAUCCAUGCAAAACUGA | PTEN,ATXN1,TGFBR2, BMPR2,HIPK1 |
| miR-31-5p | -1.55 | Downregulated | AGGCAAGAUGCUGGCAUAGCU | RHOA,SATB2,FOXP3,MMP16,RDX |
| miR-3150b-3p | -1.54 | Downregulated | CAACCUCGAGGAUCUCCCCAGC | ARL6IP1,MRVI1,RBM8A, STX6,HMGB3 |
| miR-331-3p | -1.55 | Downregulated | GCCCCUGGGCCUAUCCUAGAA | ERBB2,NRP2,DOHH,HOTAIR,E2F1 |
| miR-365a-3p | -1.56 | Downregulated | UAAUGCCCCUAAAAAUCCUUAU | CCND1,CDC25A,BCL2,KRAS,BAX |
| miR-365b-3p | -1.56 | Downregulated | UAAUGCCCCUAAAAAUCCUUAU | SNRK,YRDC,MYLIP,UBB, MCOLN2 |
| miR-5196-3p | -1.51 | Downregulated | UCAUCCUCGUCUCCCUCCCAG | H3F3C,TXNIP,GNAS,TECPR2,H3F3B |
| miR-664a-3p | -1.54 | Downregulated | UAUUCAUUUAUCCCCAGCCUACA | SNRPB2,OLIG3,ZNF667, ZBTB34,RAN |
| miR-873-3p | -1.59 | Downregulated | AGACUGGUGAGUUCCCGGGAA | HES7,DCAF7,ABCC5,TMED7,ZNF611 |

**Supplementary Table** **4.** Differently expressed miRNAs in KSHV seropositive Kaposi’s sarcoma patients v.s KSHV seronegative Kaposi’s sarcoma patients

**miRNAs Fold Change miRNAStatus miRNAsequence Target Gene**

**(FC) Value**

| let-7a-5p | 1.53 | Upregulated | UGAGGUAGUAGGUUGUAUAGUU | NF2,KRAS,HMGA2,CDK6,DICER1 |
| --- | --- | --- | --- | --- |
| let-7e-5p | 1.50 | Upregulated | UGAGGUAGGAGGUUGUAUAGUU | IGF1R,HMGA2,CCND1,WNT1, MYCN |
| miR-126-5p | 1.61 | Upregulated | CAUUAUUACUUUUGGUACGCG | SPRED1,SLC45A3,PTPN7,MYC, ADAM9 |
| miR-130a-3p | 1.62 | Upregulated | CAGUGCAAUGUUAAAAGGGCAU | HOXA5,PPARG,ATXN1,MEOX2, KLF4 |
| miR-134-5p | 1.57 | Upregulated | UGUGACUGGUUGACCAGAGGGG | MAGI2,KRAS,ITGB1,STAT5B, PUM2 |
| miR-139-3p | 2.10 | Upregulated | UGGAGACGCGGCCCUGUUGGAGU | LRPPRC,THY1,BARHL1,MTDH, FOXC1 |
| miR-1908-5p | 1.51 | Upregulated | CGGCGGGGACGGCGAUUGGUC | NKIRAS2,APOE,DNAJA4,ATP6, VGF |
| miR-196a-5p | 2.68 | Upregulated | UAGGUAGUUUCAUGUUGUUGGG | HOXC8,BACH1,CDKN1B,ANXA1,HOXA7 |
| miR-23a-5p | 1.55 | Upregulated | GGGGUUCCUGGGGAUGGGAUUU | CHD4,WNT7B,GSR,ATF1,HDGF |
| miR-3188 | 1.62 | Upregulated | AGAGGCUUUGUGCGGAUACGGGG | ADM,ESPL1,EGF,CTC1,ACTG1 |
| miR-323a-3p | 1.64 | Upregulated | CACAUUACACGGUCGACCUCU | SMAD2,SMAD3,WDR45B,MYLIP,ZXDA |
| miR-3607-3p | 1.67 | Upregulated | ACUGUAAACGCUUUCUGAUG | MIDN,GIGYF1,SMIM15,USP42, FAM3C |
| miR-3613-5p | 1.53 | Upregulated | UGUUGUACUUUUUUUUUUGUUC | ANP32B,H3F3C,VPS13B, MYO10,MTF2 |
| miR-382-5p | 1.67 | Upregulated | GAAGUUGUUCGUGGUGGAUUCG | PTEN,YBX1,MXD1,NFIA,UBB |
| miR-411-5p | 1.67 | Upregulated | UAGUAGACCGUAUAGCGUACG | GRB2,PLEKHA1,PGAM5,KPNA5,ZYG11B |
| miR-432-5p | 1.56 | Upregulated | UCUUGGAGUAGGUCAUUGGGUGG | MECP2,RCOR1,NES,BLCAP, LRPPRC |
| miR-4433b-3p | 1.78 | Upregulated | AUGUCCCACCCCCACUCCUGU | DDX39B,ABCF2,UNK,RAD21, ARF1 |
| miR-4448 | 1.54 | Upregulated | GGCUCCUUGGUCUAGGGGUA | CCDC88A,MRC1,CSNK1E,SEPT8,MSN |
| miR-493-5p | 1.64 | Upregulated | UUGUACAUGGUAGGCUUUCAUU | DDX55,SULT1B1,RAB5C,MAP7, BDH1 |
| miR-548ap-5p | 1.52 | Upregulated | AAAAGUAAUUGCGGUCUUU | ANKEF,PTBP2,SVOP,INHBA, CDK4 |
| miR-548au-5p | 1.60 | Upregulated | AAAAGUAAUUGCGGUUUUUGC | ANKEF1,PTBP2,SVOP,INHBA, CDK4 |
| miR-548j-5p | 1.52 | Upregulated | AAAAGUAAUUGCGGUCUUUGGU | ANKEF1,PTBP2,SVOP,INHBA, CDK4 |
| miR-629-5p | 1.59 | Upregulated | UGGGUUUACGUUGGGAGAACU | HNF4A,TRIM33,HIST1H2BE, ZBTB47,RSRC2 |
| miR-758-3p | 1.50 | Upregulated | UUUGUGACCUGGUCCACUAACC | TLR7,TLR3,GRPEL2,SEC24A, HMGA1 |
| miR-98-5p | 1.62 | Upregulated | UGAGGUAGUAAGUUGUAUUGUU | HMGA2,PGRMC1,E2F2,MYC, TUSC2 |
| miR-3150b-3p | -2.08 | Downregulated | CAACCUCGAGGAUCUCCCCAGC | ARL6IP1,MRVI1,RBM8A,STX6, HMGB3 |
| miR-3158-3p | -1.75 | Downregulated | AAGGGCUUCCUCUCUGCAGGAC | ZBTB39,DDX6,CDK2,CTC1, XRCC6 |
| miR-3180 | -1.81 | Downregulated | UGGGGCGGAGCUUCCGGAG | POU3F3,TULP1,H2AFX,SLC10A7,PCGF3 |
| miR-3180-3p | -1.81 | Downregulated | UGGGGCGGAGCUUCCGGAGGCC | POU3F3,TULP1,H2AFX,SLC10A7,PCGF3 |
| miR-345-5p | -1.50 | Downregulated | GCUGACUCCUAGUCCAGGGCUC | CDKN1A,ABCC1,NTRK3,PANK1,DAZAP2 |
| miR-365a-3p | -1.68 | Downregulated | UAAUGCCCCUAAAAAUCCUUAU | CCND1,CDC25A,BCL2,KRAS,BAX |
| miR-365b-3p | -1.68 | Downregulated | UAAUGCCCCUAAAAAUCCUUAU | SNRK,YRDC,MYLIP,UBB, MCOLN2 |
| miR-3690 | -2.33 | Downregulated | ACCUGGACCCAGCGUAGACAAAG | CCDC71L,STX1B,RAF1,RIMS4, ZNF486 |
| miR-3909 | -1.75 | Downregulated | UGUCCUCUAGGGCCUGCAGUCU | ADRB1,NHS,ZMAT3,TRIB1,RB1 |
| miR-451a | -1.75 | Downregulated | AAACCGUUACCAUUACUGAGUU | MIF,BCL2,RAB14,AKT1,MMP2 |
| miR-4732-3p | -1.53 | Downregulated | GCCCUGACCUGUCCUGUUCUG | GOLGA8A,REST,MAT2A, SEMA4C,SESN2 |
| miR-5196-3p | -1.50 | Downregulated | UCAUCCUCGUCUCCCUCCCAG | H3F3C,TXNIP,GNAS,TECPR2, H3F3B |
| miR-532-3p | -1.59 | Downregulated | CCUCCCACACCCAAGGCUUGCA | ERCC1,GAN,MFN2,F2, TMEM119 |
| miR-618 | -2.39 | Downregulated | AAACUCUACUUGUCCUUCUGAGU | STRN,MBD2,DVL3,ZNF529, IFNAR1 |
| miR-6503-5p | -1.55 | Downregulated | AGGUCUGCAUUCAAAUCCCCAGA | PAGR1,MAPT,ELK1,PDPK1, BCL2L14 |
| miR-6718-5p | -1.60 | Downregulated | UAGUGGUCAGAGGGCUUAUGA | SEPT2,HIST2H4A,LYRM2, NLGN4X,VEZF1 |
| miR-941 | -1.50 | Downregulated | CACCCGGCUGUGUGCACAUGUGC | KDM6B,ZNF431,HSPA6,TRPS1, CEP19 |
